# Twenty-Five Years of Structural Parvovirology

**DOI:** 10.3390/v11040362

**Published:** 2019-04-20

**Authors:** Mario Mietzsch, Judit J. Pénzes, Mavis Agbandje-McKenna

**Affiliations:** Department of Biochemistry and Molecular Biology, Center for Structural Biology, The McKnight Brain Institute, University of Florida, Gainesville, FL 32610, USA; mario.mietzsch@ufl.edu (M.M.); judit.penzes@ufl.edu (J.J.P.)

**Keywords:** parvovirus, densovirus, single stranded DNA virus, X-ray crystallography, Cryo-EM, antibody interactions, receptor interactions

## Abstract

Parvoviruses, infecting vertebrates and invertebrates, are a family of single-stranded DNA viruses with small, non-enveloped capsids with T = 1 icosahedral symmetry. A quarter of a century after the first parvovirus capsid structure was published, approximately 100 additional structures have been analyzed. This first structure was that of Canine Parvovirus, and it initiated the practice of structure-to-function correlation for the family. Despite high diversity in the capsid viral protein (VP) sequence, the structural topologies of all parvoviral capsids are conserved. However, surface loops inserted between the core secondary structure elements vary in conformation that enables the assembly of unique capsid surface morphologies within individual genera. These variations enable each virus to establish host niches by allowing host receptor attachment, specific tissue tropism, and antigenic diversity. This review focuses on the diversity among the parvoviruses with respect to the transcriptional strategy of the encoded VPs, the advances in capsid structure-function annotation, and therapeutic developments facilitated by the available structures.

## 1. Introduction

**Figure 1:**
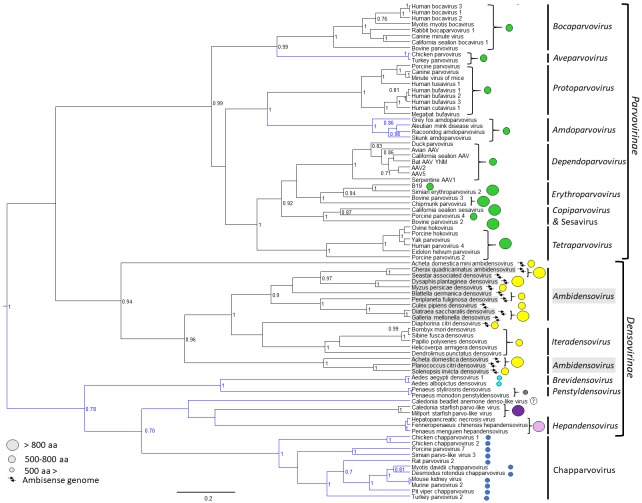
Evolutionary relationships of members of family *Parvoviridae* based on the conserved NS1 tripartite helicase domain. Branches of lineages highlighted in blue indicate the absence of a phospholipase A_2_ (PLA_2_) domain in the minor capsid viral protein, VP1. Capsid protein encoding gene homology is mapped as circles of different colors, where same colored circles indicate homologous genes (homology search defined, without the incorporation of the PLA_2_ sequence, as whether a protein sequence gives a hit out of targeted 5000 sequences at an expectation value of 100 by the BlastP algorithm of the NCBI Blast application [2]). The size of the circle indicates the size of VP1 based on the scale to the left.

The *Parvoviridae* are linear, single-stranded DNA packaging viruses with genomes of ~4 to 6 kb. They have a large host spectrum, spanning members of the phylum Cnidaria to amniote vertebrates. Currently the *Parvoviridae* is divided into two subfamilies based on their ability to infect either vertebrates or invertebrates [1]. Viruses infecting vertebrate and invertebrate hosts are assigned to *Parvovirinae* and *Densovirinae* subfamilies, respectively, although the monophyly of the latter is questioned due to the diversity of members, and new emerging vertebrate viruses close to the *Densovirinae* may require a new subfamily (Figure 1).

**Figure 2:**
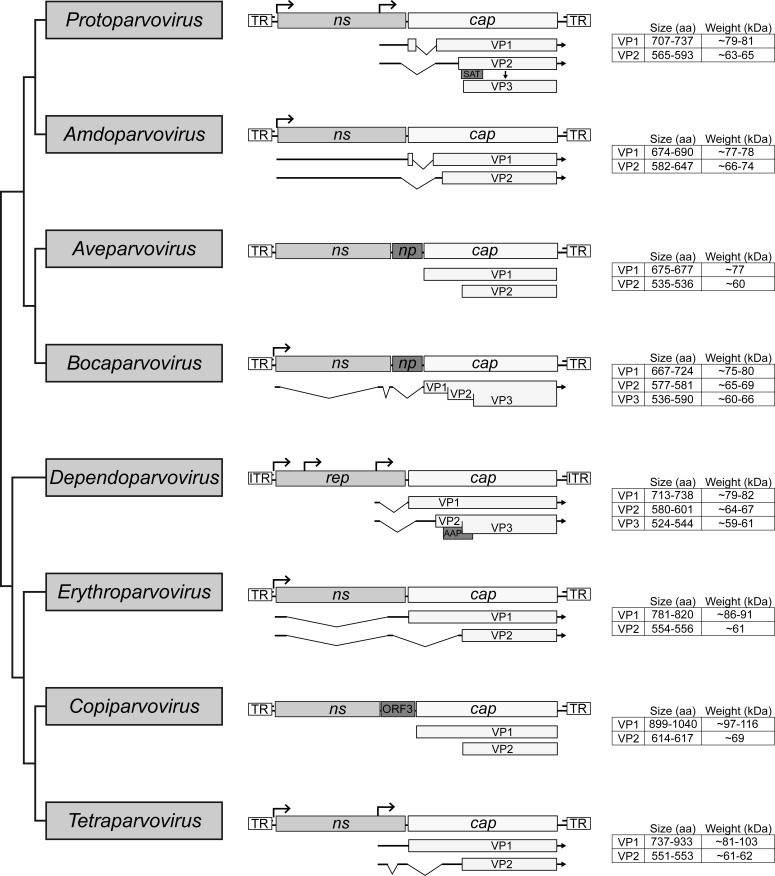
Cladogram of the subfamily *Parvovirinae*. The eight genera are shown. The general genome organization of each genus is shown in the middle with their ORF. The non-structural (NS) protein expressing genes *ns* or *rep* are simplified and only the ORF is shown. Below the *cap* ORF the transcripts for the expression of the individual VP are shown. On the right side the size and weight of the VPs are given. Note that the transcription profiles of the Aveparvovirus and Copiparvovirus genera have not been determined, and thus the sizes of the VPs are based on in silico predictions.

**Figure 3:**
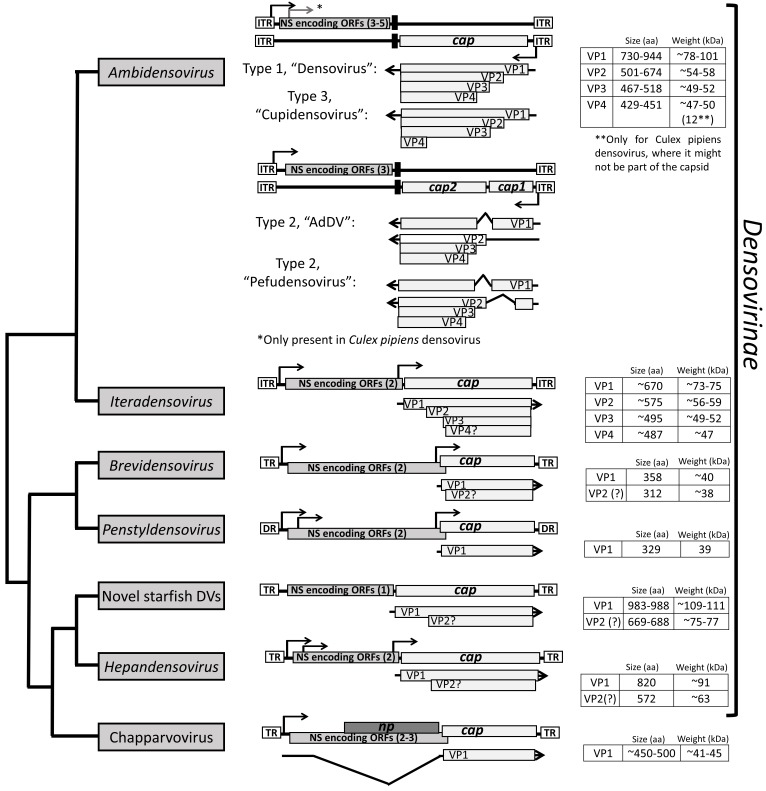
Cladogram of denso- and chapparvoviruses. The general genome organization and capsid protein expression strategy are shown. The *ns* genes are simplified and only the ORF is shown. The transcription strategy of members of genus *Hepandensovirus*, as well as of the new, unclassified starfish densoviruses, have not been determined, thus the sizes of the VPs are based on in silico predictions.

Parvovirus virions possess small non-enveloped capsids with a diameter of 200 to 280 Å [3,4,5,6,7,8,9]. Their T = 1 icosahedral capsids are assembled from 60 viral proteins (VPs) encoded from the right-hand side open reading frame (ORF) (Figure 2 and Figure 3). This ORF, also known as *cap*, encodes up to four different VPs, depending on genus, of varying length, which all share a common C-terminal region [1]. Generally, the smallest VP, which comprises the common C-terminal region, is expressed at a higher rate compared to the larger VP forms and is, therefore, considered the major VP. The larger, less abundant VPs are N-terminal extended forms that contain regions important for the viral life cycle. Among these are a phospholipase A_2_ (PLA_2_) domain, a calcium-binding domain, and nuclear localization signals that are highly conserved in some genera [10,11,12,13]. The larger VPs are also incorporated into the capsid, albeit at low copy number, with the common C-terminus responsible for assembling the parvoviral capsid. In the different parvoviruses, the shared VP region varies between ~40 to 70 kDa in size (Figure 2 and Figure 3). This review focuses on the transcription mechanism, and the sequence- and structure-based homology of the parvovirus VPs, as well as the characteristic features of the parvoviral capsids. We also discuss structure-function annotation and the use of structure to guide the development of gene delivery vectors.

## 2. Parvovirinae

The Parvovirinae is subdivided into eight genera: *Amdoparvovirus*, *Aveparvovirus*, *Bocaparvovirus*, *Dependoparvovirus*, *Erythroparvovirus*, *Copiparvovirus*, *Protoparvovirus*, and *Tetraparvovirus* (Figure 1 and Figure 2) [1]. As the genus name suggests, the dependoparvoviruses require helper virus functions for replication [14,15,16,17,18]. All other genera contain members capable of autonomous replication. The *Parvovirinae* viral genomes contain two or three ORFs (Figure 2). The left ORF, the ns or rep gene, encodes a series of regulatory proteins that are indispensable for viral replication. Due to its higher level of conservation, this gene is used for the classification of parvoviruses into different genera (Figure 1). The right ORF, the cap gene, encodes up to three VPs that assemble the capsid (Figure 2) [1]. In addition, multiple genera express smaller regulatory proteins, such as nucleoprotein 1 (NP1) by the *Aveparvovirus* and *Bocaparvovirus* encoded near the middle of their genome, or the assembly activating protein (AAP) by the *Dependoparvovirus*, and the small alternatively translated (SAT) and non-structural protein 2 (NS2) by the *Protoparvovirus*, encoded within alternative reading frames of the main ORFs.

### 2.1. Expression of the Parvovirinae VPs Utilizes Different Transcription Strategies

The VPs of the *Parvovirinae* are encoded in the same orientation as the *ns* or *rep* gene. Members of the *Amdoparvovirus*, *Bocaparvovirus*, and *Erythroparvovirus* use the same promoter for the expression of the NSs and VPs (Figure 2). The *Dependoparvovirus*, *Protoparvovirus*, and *Tetraparvovirus* utilize an additional promoter located at the 3’ end of the *ns*/*rep* gene for the expression of the *cap* ORF [19,20]. The transcription profiles for *Aveparvovirus* and *Copiparvovirus* are unknown. For the expression of the different VPs, most *Parvovirinae* members perform alternative splicing of their transcripts or utilize alternative start codons (Figure 2) [19,20,21,22,23,24,25]. Differences in splicing efficiency and leaky scanning during translation initiation, as well as the utilization of non-canonical start codons, result in the higher expression of the smallest VP form over the larger N-terminal extended forms (Figure 2) [20,21,22,23,24,25]. The translated VPs are translocated to the nucleus, where they assemble into the 60 mer capsid [26,27]. Based on their expression levels, the minor and major capsid VPs are reported to be incorporated at ratios of 1:10 for VP1:VP2 in capsids containing two VPs, for example, human parvovirus B19, and 1:1:10 for VP1:VP2:VP3 in capsids with three VPs, such as the Adeno-associated viruses (AAVs) [28,29,30,31]. For some *Parvovirinae* members this process is assisted by additional proteins, such as the AAP of the *dependoparvoviruses* or NS2 of the *protoparvoviruses* [27,32]. The viral genome is reportedly translocated into pre-assembled empty capsids utilizing the helicase function of NS/Rep proteins [33]. For some protoparvoviruses, proteolytic cleavage of VP2 following DNA packaging removes ~20 to 25 amino acids from the N-terminus to create VP3 (Figure 2) [34,35,36,37].

### 2.2. Parvovirinae Genera Display Distinct Capsid Surface Morphologies

The first structure of a parvovirus, that of wild type (wt) canine parvovirus (CPV), a member of *Protoparvovirus*, was published in 1991, with the VP structure coordinates deposited in 1993, a quarter of a century ago [6,38]. Since then numerous other capsid structures, >100, have been determined for the protoparvoviruses, as well as members of three other (of the eight) *Parvovirinae* genera, including complexes with receptors or antibodies (Table 1, Section 2.5, and Section 2.6). The structures of capsids alone include those of wt as well as variants. The studies have primarily utilized X-ray crystallography, and in recent years increasingly cryo-electron microscopy and 3-dimensional image reconstruction (cryo-EM) as the method began to generate atomic resolution structures. The most capsid (no ligand) structures determined have been for the dependoparvoviruses and protoparvoviruses, for which more than 20 structures are available, followed by four for the bocaparvoviruses, and one for *Erythroparvovirus* (Table 1).

**Figure 4:**
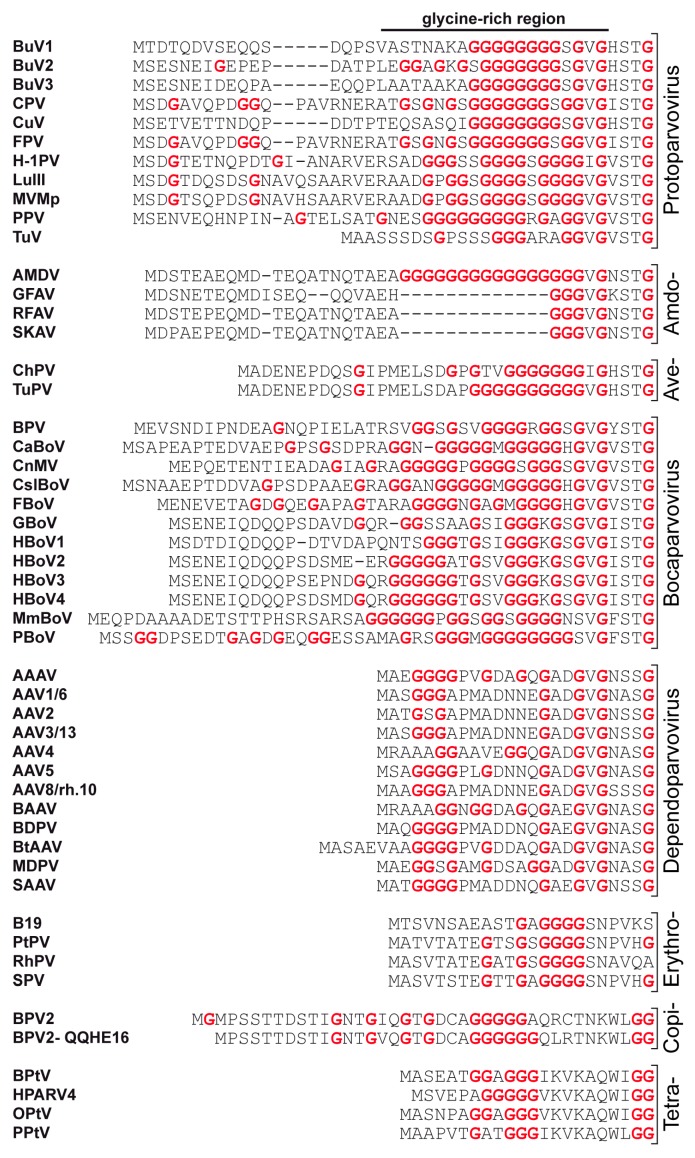
The N-termini of the major VPs of the *Parvovirinae*. For each genus a selection of available VP sequences are shown for the N-terminal 20–50 amino acids. All glycine residues are shown in red.

**Figure 5:**
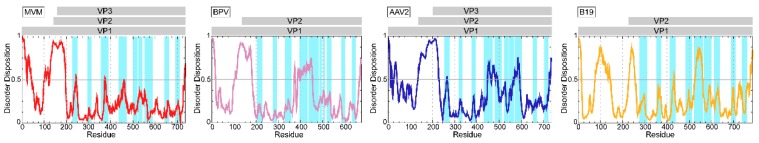
Disorder prediction for type members MVMp (red), BPV (pink), AAV2 (blue), and Parvovirus B19 (orange) VP1 by the PONDR_fit application [72]. Regions above 0.5 on the Y-axis are predicted to be disordered. Gray line drawings above the images indicate the approximate positions of the VPs. The regions highlighted in light blue indicate the locations of the surface exposed loops, the tops of which are defined as variable regions.

In all the structures, the N-terminal regions of the larger VP (e.g., VP1u), as well as the N-terminal 20-40 amino acids of the major VP, are not resolved. The N-termini of the larger VPs are believed to be located on the inside of the capsid and were shown not to affect the overall capsid structure [70]. The inability to determine the structure of these N-termini is likely due to their flexibility and their low copy numbers within the capsid. The exception to this was human parvovirus B19, for which low resolution cryo-EM maps showed density interpreted as the VP2 N-termini [71]. The flexibility resulting in the N-termini disorder arises from a glycine-rich N-terminal region present in most *Parvovirinae* (Figure 4). Furthermore, the low copy number of the minor VPs or a different positioning of the N-terminus of the different VPs is incompatible with the icosahedral averaging applied during structure determination. A disorder prediction indicates that the glycine-rich stretch is highly disordered in all analyzed *Parvovirinae*, while the VP1u and the overlapping C-terminal VP region are generally more ordered (Figure 5).

The cluster of glycines and associated flexibility likely serve to enable the externalization of VP1u for the PLA_2_ function contained within [10]. Consistently, in reported *Parvovirinae* structures, the first ordered N-terminal residue of the overlapping VP region is located after the glycine-rich region, and inside the capsid under the only channel in the capsid. This channel, located at the icosahedral 5-fold axis, connects the inside and outside of the capsid (see below) [38,39,47,73,74]. The remainder of the VP is ordered to the C-terminus in all *Parvovirinae* structures determined to date.

**Figure 6:**
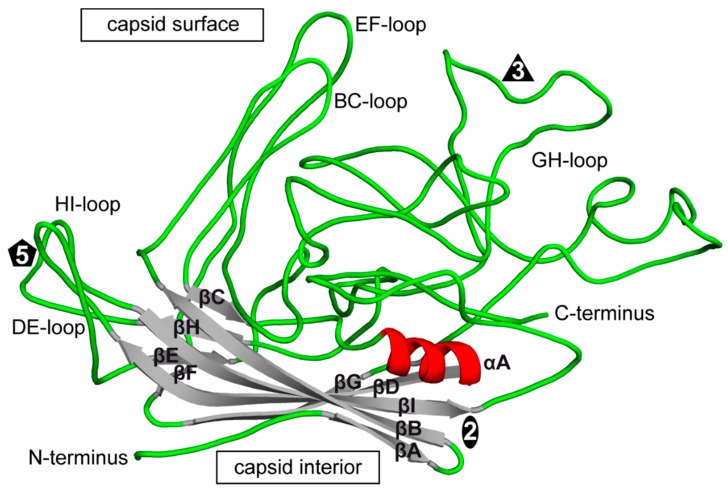
The structure of a VP monomer of CPV (PDB-ID: 2CAS). A cartoon ribbon diagram is shown. The beta strands (βA to βI, gray), α-helix A (red), interconnecting surface loops (with all secondary structure elements removed, green), and the N- and C-terminus are indicated. The approximate icosahedral 2-fold, 3-fold, and 5-fold axis are indicated by an oval, triangle, and pentagon, respectively. This image was generated using PyMOL [77].

**Figure 7:**
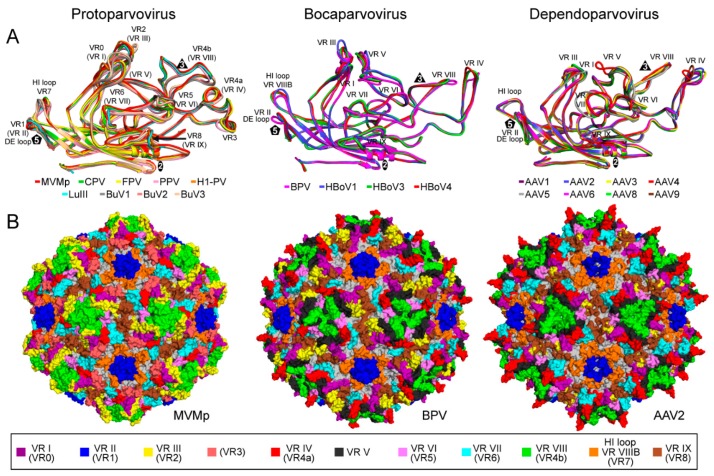
The VRs of the *Parvovirinae*. (**A**) Structural superposition of VP monomers from different members of *Protoparvovirus* (left), *Bocaparvovirus* (center), and *Dependoparvovirus*. Individual colors for the ribbons are as indicated. The VRs: VR-I to VR-IX (or VR0 to VR8 for the protoparvoviruses), the DE, and HI loops are shown. (**B**) Location of the VRs, colored as indicated, on the capsid surface of MVMp as an example for *Protoparvovirus* (left), BPV for *Bocaparvovirus* (center), and AAV2 for *Dependoparvovirus* (right). The figures were generated using PyMOL [77].

The CPV structure confirmed the icosahedral nature of the parvovirus capsids with sixty VPs assembling one capsid via 2-, 3-, and 5-fold symmetry related interactions [6,38,75]. The *Parvovirinae* VP structures display significant similarity despite low sequence identities (Table 2). The ordered VP monomer region consists of a core eight-stranded (βB to βI) anti-parallel β-barrel motif, also known as a jelly roll motif, with a BIDG sheet that forms the inner surface of the capsid (Figure 6) [76]. This β-barrel is conserved in all parvoviral capsid structures determined to date, as has been reported for many other viruses. In addition, a βA strand that runs anti-parallel to the βB strand of the BIDG sheet, and a conserved helix, αA, located between strands βC and βD, are also part of conserved *Parvovirinae* core structure (Figure 6). Loops inserted between the β-strands of the β-barrel form the surface of the capsid. These loops are named after the β-strands that they connect, for example, the DE loop connects the βD and βE strands. The GH loop that connects the βG and βH strands is the largest surface loop consisting of multiple sub-loops (Figure 6). The surface loops contain the highest amino acid sequence and structural diversity among members of the same genus and between different parvoviruses in general (Figure 7). Differences at the apexes of these loops are termed variable regions (VRs), defined as two or more amino acids with Cα positions greater than 1 Å apart (for the dependoparvoviruses) [60] or 2 Å apart (for the other *Parvovirinae*) [46] when their VPs are superposed. For the VPs of protoparvoviruses, bocaparvoviruses, and dependoparvoviruses nine to ten VRs have been defined (Figure 7).

**Figure 8:**
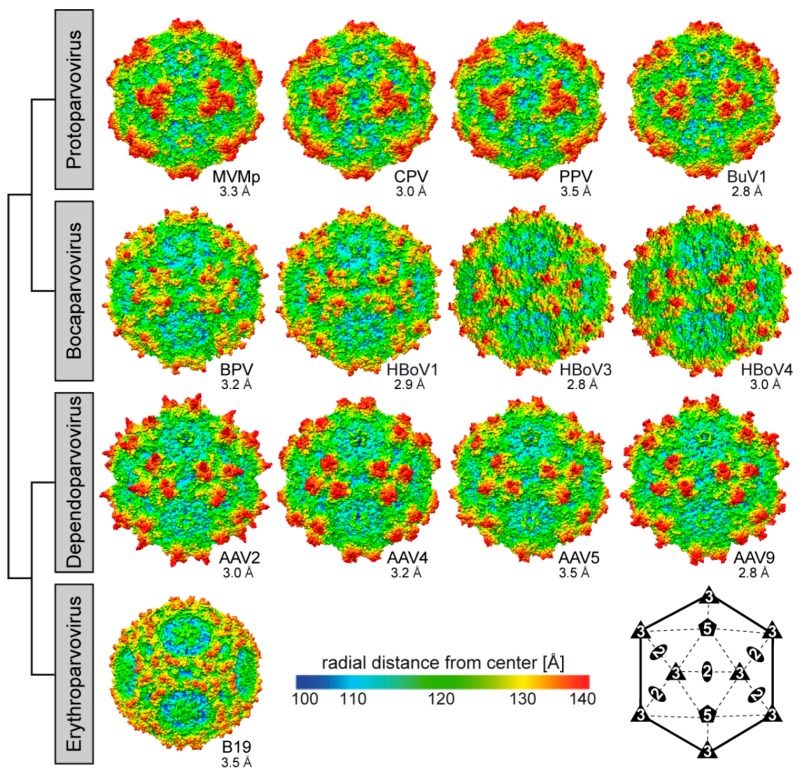
Capsid structures of the *Parvovirinae* subfamily. A selection of capsid structures is shown for *Protoparvovirus*, *Bocaparvovirus*, *Dependoparvovirus*, and *Erythroparvovirus*. The capsid surfaces are viewed down the icosahedral 2-fold axes and are colored according to radial distance from the particle center (blue to red), as indicated by the scale bar. The capsid images were generated using Chimera [78]. In the lower right hand side, a symmetry diagram illustrating the positions of the icosahedral symmetry axes on the capsid surfaces is shown.

The locations of the VRs as well as the overall structure of the VP monomer within each genus and among the *Parvovirinae* are similar despite sequence identities as low as 15% (Table 2, Figure 7). The different VR conformations create distinct genus level morphologies for the capsids, although the capsids have the same overall characteristic features (Figure 8). These include a channel at the icosahedral 5-fold symmetry axes, assembled by five DE loops, surrounded by a depression, described as canyon-like, lined by the HI loop located above the neighboring VP’s βCHEF strands. The channel at the 5-fold axes connects the interior of the capsid to the exterior and is believed to play an important role in most parvoviruses during the viral replication cycle by serving as the route of viral genome packaging, genome release, and the externalization of VP1u for its PLA_2_ function [70,73]. Secondly, protrusions are located at or surrounding the icosahedral 3-fold axes assembled by loop/VR contributions from two or three VP monomers depending on genus (Figure 8). Variable regions IV, VR-V, and VR-VIII from two 3-fold related VP monomers contribute to the three separate protrusions of the dependoparvoviruses (Figure 7 and Figure 8). Similarly, VR-IV, VR-V, and VR-VIII contribute to the 3-fold protrusions in the bocaparvoviruses, along with VR-I generating several dispersed peaks. In B19, two separate protrusions surround the icosahedral 3-fold axes (Figure 8). One of these protrusions is formed by VR-I and VR-III, the other by VR-VIII. However, the B19 structure lacks 13 residues within VR-V, which is located between both protrusions [4]. This region (aa 528–540, VP1 numbering) is predicted to be highly disordered (Figure 5) and could potentially merge both protrusions. In the animal protoparvoviruses, where the VRs are defined by Arabic numerals, VR0 (VR-I in the dependoparvoviruses), VR2 (VR-III), and VR4b (VR-VIII) form the single pinwheel 3-fold protrusions (Figure 7 and Figure 8) [46,50,60]. In contrast, a deletion in VR4b near the 3-fold symmetry axis results in separated 3-fold protrusions for the bufaviruses (BuVs) (Figure 7 and Figure 8) [39]. Within and among genera, the shape and size of the 3-fold protrusions vary because of sequence length and conformational loop differences. The variable surface loops at the 3-fold are reported to mediate the interactions of parvoviruses with different host factors, including receptors and antibodies (see Section 2.6 and Section 2.7) [74]. Thirdly, a second depression is located at the 2-fold symmetry axes of the capsid (Figure 8). The floor of the depression is lined by a conserved (within genus) stretch of residues C-terminus of the βI strand. The shape of the depression, however, is variable in depth and width within and between genera (Figure 8) due to differences in side-chain orientations. The 2- and 5-fold depressions are separated by a raised capsid region, termed the 2-/5-fold wall, which displays structural variability among the *Parvovirinae* due to conformational differences in VR-VII and VR-IX. The 2-fold depression serves as a site for glycan receptor interaction for members of the protoparvoviruses, while the 2-/5-fold wall serves to bind receptors as well as antibodies for different genera (see Section 2.6 and Section 2.7) [74].

Unique to the structure of bocaviruses is a “basket-like” feature underneath the 5-fold axis that extends the channel further into the interior of the capsid [5,54]. The basket arises from density located at the N-terminus of the observable VP structure and includes parts of the glycine-rich region [54]. This ordered density under the 5-fold channel poses a problem with the proposed infection mechanism. The hypothesis is that at low pH conditions, similar to the environment in the late endosome, structural rearrangements of the basket occur that open up the 5-fold channel for VP1u externalization for its PLA_2_ function. Interestingly, the structures of AAV8, CPV, and feline panleukopenia virus (FPV) determined at low pH conditions show structural changes at residues and capsid surface loops, although the 5-fold channel was not reported to be altered [42,64].

### 2.3. Nucleotides Are Ordered Inside Parvovirinae Capsids Despite Lack of Icosahedral Symmetry

The ordering of nucleotides (nts) inside the capsids of some *Parvovirinae* is observed despite the lack of adherence of the single copy of the packaged genome or reporter gene to icosahedral symmetry. This has been observed for virus-like particles (VLPs) and DNA packaged (full) members of the dependoparvoviruses and full protoparvoviruses. A conserved pocket under the 3-fold symmetry axis shows the ordering of one or two nts for multiple AAV serotypes [59,60,63,64,68,69]. Low pH conditions reduced the ordered DNA density [64]. The loss of the nt density at pH 4.0 suggested loss of capsid-DNA interaction that serves as one of the steps leading to release of the genome from the capsid following endosomal trafficking during infection [64]. The ordering of an nt in VLPs in absence of Rep protein suggests that capsid assembly may require nucleation by an nt for the dependoparvoviruses [63]. For the protoparvoviruses, large stretches of ordered DNA, 11 nt in CPV [6], 19 nt in minute virus of mice strain i (MVMi) [49,50], and 10 nt in H-1PV [46], have been reported. The ordering of more nt compared to the dependoparvoviruses may be due to the packaging of only the negative sense genome in the protoparvoviruses, while the AAV packaging both polarities. The ordered protoparvoviruses nts are located within a pocket inside the capsid adjacent to the icosahedral 2-fold axes in all the viruses. This was suggestive of a recognition motif, but a search through the wt genome of CPV identified matches only when 2–3 nt mismatches were allowed [79]. Thus, the role of this organized DNA beyond a common binding site in the *Parvovirinae* is yet to be determined.

### 2.4. The Structure of Capsid Variants Provide Insight into Function

As listed in Table 1, the structures of several variants have been determined for the *Parvovirinae*. The aims of these studies were structure-function understanding of observed biological phenotypes. The majority of variant structures studied are for AAV2, CPV, and MVM, which are among the best functionally characterized members of this subfamily. Currently, the highest resolution virus structure is that of an AAV2 variant with the leucine at position 336 (VP1 numbering) mutated to a cysteine, AAV2-L336C, at 1.9 Å, obtained by cryo-EM [57]. This mutant has a genome packaging defect and altered VP1u externalization properties [80]. A comparison of this structure to wt AAV2 identified a destabilization of the VP N-terminus inside the capsid and the widening of the base of the 5-fold channel in the variant [57]. This observation supports previous claims that the 5-fold region functions as a portal of genome packaging and VP1u externalization, and that the correct arrangement of the residues in the channel plays a crucial role in these functions. The structure of a similar variant with the equivalent leucine mutated to a tryptophan, was analyzed for MVM, MVM-L172W (VP2 numbering), by X-ray crystallography (Table 1) [51]. This variant was also reported to have a DNA packaging defect and altered VP1u externalization dynamics phenotype [81]. In the MVM-L172W structure, the tryptophan blocked the channel and also induced a reorganization of the N-terminus of VP2 [51]. This suggests that different perturbations in the same structural location can result in a similar inhibitory phenotype. A second AAV2 variant, AAV2-R432A, also characterized the determinant of another genome packaging defective virus resulting from a single residue change [82]. In the wt AAV2 structure, this residue is located within the capsid, neither on the inside nor the outside surface, and at the 3-fold axes. Its side-chain interacts with the main-chain of a surface loop [56]. The AAV2-R432A structure, also determined by cryo-EM, detailed the propagation of capsid destabilization to distant sites from R432, including the rearrangement of the βA strand and movement of residue side-chains at the base of the 5-fold channel inside the capsid. The capsid was also less thermally stable than wt AAV2 [56]. Together, the data suggested that the structure rearrangements and destabilization resulted in packaging incompetence. The crystal structures of CPV variants, CPV-N93D, CPV-N93R, and CPV-A300D (VP2 numbering) in Table 1 were studied to understand the juxtaposition of amino acids controlling tissue tropism and antigenicity [41,42,43]. These residues form the footprint of the transferrin receptor and several antibody epitopes [43,83,84]. Furthermore, these studies showed that amino acid determinants could be localized far apart but function together [41,85].

### 2.5. Capsid-Receptor Complex Structures

The role of the parvoviral capsid is to protect the packaged genome and to deliver it to the nucleus of the target cells for the next replication cycle. For the *Parvovirinae,* glycan receptors, for example terminal sialic acid (SIA), galactoses, heparan sulfate proteoglycans, P-antigen, and various proteins, including AAVR, transferrin, laminin, fibroblast growth factor receptor, hepatocyte growth factor receptor, and epidermal growth factor receptor, serve as receptors [83,86,87,88,89,90,91,92,93,94]. The structures of several of the ligands bound to their capsids have been determined. Presently, the number of published capsid-receptor complex structures at atomic resolution is low (Table 3), but due the recent advancement of cryo-EM this number will increase. In capsid-glycan complex structures, the receptors are bound at or around the 3-fold protrusion (AAV2:heparin, AAV-DJ:heparin) [95,96,97], at the center of the 3-fold symmetry axis (AAV3:sucrose octasulfate, AAV5:SIA) [98,99], at the base of the 3-fold protrusion (AAV1:SIA) [100], and in a pocket near the 2-fold symmetry axis (MVM:SIA) [101]. The MVM-SIA structure was the first to be determined for a receptor complex. Similar to glycans, cellular protein receptors can bind symmetrical to the capsid, as has been shown recently for the PKD2 domain of AAVR to the capsid of AAV2 that interacts with the 3-fold protrusion and the 2-/5-fold wall [102]. However, larger protein receptors might bind with lower copy number to the capsid surface as observed for the transferrin receptor to CPV capsids [83]. The CPV-transferrin complex was the first structure determined for a protein receptor on a parvovirus capsid. The transferrin footprint is located on the 2-/5-fold wall and includes residues 93, 299, and 301 [83,85].

### 2.6. Capsid-Antibody Complex Structures

**Figure 9:**
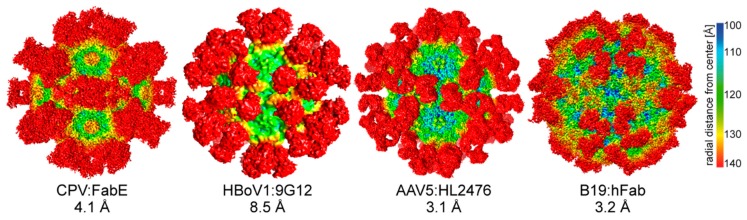
*Parvovirinae*-antibody complex structures. The highest resolution complex structures available for *Protoparvovirus*, *Bocaparvovirus*, *Dependoparvovirus*, and *Erythroparvovirus* are shown. The cryo-EM density maps are viewed down the icosahedral 2-fold axis and are colored according to radial distance from the particle center (blue to red), as indicated by the scale bar. The FAbs decorating the capsid surface are in red. The FAbs bind across the icosahedral 2-fold (e.g., CPV:FAbE), the 2-/5-fold wall (HBoV1:9G12), the 3-fold (AAV5:HL2476), and 5-fold depression (B19:hFab). The images were generated using Chimera [78]. (CPV: EMD-6629, B19: EMD-9110).

The infection by members of the *Parvovirinae* elicits the host immune response, resulting in both neutralizing and non-neutralizing antibodies raised against their capsids. In the human population, the seroprevalence against different members of the *Parvovirinae* can be high. For example, while the seroprevalence varies in different regions of the world, up to 80% of adults have antibodies against B19 [103]. Similar percentages of positivity exist against capsids of different AAV serotypes [104], up to 70% against the human bocaviruses [105], up to 85% against the different BuVs [106], and up to 40% against human parvovirus 4 [107]. In order to understand the antigenicity of these viruses, the structures of capsid antibodies (whole IgG or FAb) have been determined using cryo-EM (Table 4, Figure 9). The resolutions of these structures range from 23 to 3.1 Å (Table 4). The lower resolution structures are sufficient for the identification of epitopes on the capsid surface to confirm by mutagenesis. The higher resolution structures, e.g., AAV5-HL2476 and B19-human antibody complex, enables analysis of the capsid–antibody interaction for direct identification of contact residues on both sides, namely the capsid surface and residues in the CDRs of the antibody if the antibody sequence is available [108]. The complex structures have shown that almost the entire surface of these capsids can be bound by antibodies, with epitopes across the 2-fold, the 2-/5-fold wall, 3-fold protrusions, and around the 5-fold channel (Table 4, Figure 9). This information can inform the engineering of the capsids variants (Section 2.7), the development of vaccines against pathogenic members of the *Parvovirinae*, and for a better understanding of the viral life cycles, as some antibodies do not neutralize infection or can even further enhance their infection, as reported for B19 and for Aleutian mink disease parvovirus [109,110].

### 2.7. Engineering of Parvovirus Capsids to Create Biologics

The development of *Parvovirinae* members as biologics is primarily focused on the engineering of capsids of members that can be used as viral vectors in gene delivery applications, such as the AAVs [121], or more recently also bocaviral vectors [122]. For such vectors, a transgene expression cassette is packaged into the capsids instead of the wt viral genome [123]. These vectors are used to infect a desired target tissue to achieve long-term expression of the transgene to correct monogenetic diseases.

Following the discovery that AAV capsids become phosphorylated at tyrosine residues after cell entry subsequently leading to their degradation following lysine ubiquitination, reducing the transgene expression [124,125], the structural information of the AAV capsids was used to identify surface exposed tyrosines for modification [126]. Mutational analysis of these tyrosine residues to phenylalanine led to the development of engineered capsids that showed improved transduction efficiencies compared to vectors packaged into wt capsids [126]. Subsequent mutation of capsid surface serine, threonine, and lysines further improved transduction efficiency [127,128]. Another application of structure information for vector engineering is the modification of AAV capsids to escape pre-existing neutralizing antibodies utilizing the footprints mapped by cryo-EM. As mentioned above, a large percentage of the human population possesses anti-capsid antibodies against one or more AAV serotype due to natural exposures. These pre-existing antibodies bind to the capsids of administered AAV vectors and disrupt multiple steps required for successful transgene delivery, including receptor attachment, post-entry trafficking, and capsid uncoating events [129]. To circumvent these inhibitory events, different strategies have been developed, including the utilization of immunosuppressants [130,131,132,133], the utilization of alternative natural AAV capsids that are not detected by the pre-existing human antibodies [68], the use of empty capsids as decoys [134], and the structure-guided modification of the antigenic sites on the surface of the capsids [135]. For the latter strategy, the antigenic sites are identified using monoclonal antibodies, as mentioned in Section 2.6. By rational design or directed evolution, these sites can be changed to obtain new variants with escape phenotypes while maintaining infectivity [108,135,136]. While the majority of capsid engineering has been with AAVs, because of their high seroprevalence, vectors based on bocaviruses will face similar obstacles and require solutions to escape pre-existing immunity in the human population.

Another purpose for capsid engineering is the retargeting of vectors to specific receptors or tissues to restrict the broad tissue tropism of some AAV serotypes [137,138]. This can be achieved by insertion of specific targeting peptides into capsid surface loops, especially in the apexes of VR-IV and VR-VIII (Figure 7), e.g., for AAV2 variants 7m8 or r1c3 [138,139,140], directed evolution for a specific cell type [141], or structure guided approaches [142]. For some of these engineered AAVs, the structures of the modified capsids have been determined, e.g., AAV2.5, the first structure-guided in silico designed AAV gene delivery vector [58], AAV-DJ, a chimera created through random homologous recombination followed by directed evolution [67], and AAV9-L001, an AAV9 variant with a peptide lock to prevent off-target delivery [66].

## 3. Densovirinae

The *Densovirinae* encompasses members infecting exclusively invertebrates [143]. Currently, the subfamily consists of five genera, clustering into two separate lineages; *Ambi*- and *Iteradensovirus* infecting arthropods and echinoderms in the first, and *Brevi*-, *Hepan*-, and *Penstyldensovirus* infecting various arthropods, e.g., decapod crustaceans and insects in the second lineage (Figure 1) [1]. However, as the number of invertebrate-infecting parvoviruses from diverse host species has increased, the heterogeneity of the subfamily has become apparent, questioning the monophyly of prior genera, such as *Ambidensovirus* [1,13] (Figure 1). The second lineage includes additional, yet unclassified virus species. These viruses comprise the recently discovered starfish densoviruses of the species *Aster rubens* and a vertebrate-infecting parvovirus clade named Chapparvovirus after the Chiroptera-Aves-Porcine acronym based on the host species where these viruses were first discovered (Figure 1) [144]. Densoviral genomes vary in organization, unlike the subfamily *Parvovirinae*, and have a wider size range at between 3.9 and 6.3 kb. They also have either ambisense or monosense transcription. The left-hand side ORF contains the *ns* gene and expresses up to five proteins [145]. The right-hand side ORF is *cap* and encodes up to four VPs (Figure 3). All densoviruses discovered to date are capable of autonomous replication and pathogenic [1].

### 3.1. The Densovirinae Utilize Diverse Strategies for VP Expression

Densoviruses, like their vertebrate counterparts, have evolved diverse expression strategies to overcome the limitation of the coding capacity imposed by their small genome size (Figure 3) [146]. The transcription strategy has been determined for four of the five densovirus genera, and has not been experimentally derived for the *Hepandensovirus* or the new starfish densoviruses (GenBank accession numbers: MF190038 and MF190039). Overall, densoviral transcription relies more on leaky scanning than the alternative splicing utilized by the *Parvovirinae* (compare Figure 2 to Figure 3) [143]. This difference is an adaption to the host because invertebrates possess a lower percentage of alternatively spliced genes compared to vertebrates [147]. Consistently, the chapparvoviruses mentioned above (Figure 1 and Figure 3) utilize alternative splicing as the major strategy to express their VPs [148]. As for the *Parvovirinae*, the smallest VP is the one with the largest incorporation into the capsid for densoviruses.

The *Ambidensovirus* display the most variable VP expression strategies, likely because the genus currently also contains the most members. Because of their unique ambisense genome organization, the *cap* gene is located on the opposite strand relative to the *ns* gene, both driven by the furthest upstream promoter embedded in the partially double-stranded region of the ITRs (Figure 3). There are three different VP expression strategies established for this genus [1]. Members of the first group, e.g., *Galleria mellonella* densovirus (GmDV), express a minor capsid VP1 from an unspliced transcript of the p93 promoter. Three additional VPs, VP2, VP3, and VP4 (major capsid VP), are expressed by leaky scanning [7,149]. These are reported to be incorporated into the capsid at a 1:9:9:41 ratio [7]. The second group, including *Periplaneta fulliginosa* densovirus (PfDV) and *Acheta domestica* densovirus (AdDV), has a split *cap* ORF for the minor capsid VPs joined by splicing of transcripts, with VP2, VP3, and VP4 expressed from leaky scanning of the unspliced transcript in case of AdDV. This results in both VP1 and VP2 having unique N-terminal regions. These VPs are reportedly incorporated into the AdDV capsid at a 1:11:18:30 ratio. In comparison, both VP1 and VP2 are translated from spliced transcripts in PfDV (Figure 3) [8,150,151]. The third group, represented by *Culex pipiens* densovirus of *Dipteran ambidensovirus 1*, has four VPs expressed from one unspliced transcript by leaky scanning, giving rise to a VP1, VP2, VP3, and a small 12 kDa VP4, which is a minor capsid protein with approximately the same incorporation as VP1. VP2 and VP3 are reportedly equally abundant in the capsid [145].

The *Iteradensoviruses* are related to the ambidensoviruses and have a similar VP expression strategy despite packaging monosense genomes. Although the exact number of VPs expressed is unknown, they use leaky scanning from the same unspliced transcript (Figure 3) [152]. SDS-PAGE analysis of *Bombyx mori* densovirus 1 (BmDV1) show three VPs, VP1, VP2, and VP3 [153]. *Penstyl*- and *Brevidensovirus* transcribe only a single unspliced VP transcript resulting in a single VP that is among the smallest in the *Parvoviridae* at 329 aa and 358 aa, respectively [154,155]. In contrast, the *Hepandensovirus cap* ORF encodes a large VP1, e.g., with hepatopancreatic necrosis virus having a VP1 of 830 aa from the largest *Parvoviridae* genome of 6.3 kb [146,156]. The two recently discovered densoviruses from the starfish species *Aster rubens*, closely related to the *Hepandensovirus* (Figure 3), both encode the largest VPs of the family, with 983 and 988 aa (GenBank accession numbers: MF190038 and MF190039).

### 3.2. Densovirinae Capsid Structures Display Distinct Surface Morphology

In contrast to the *Parvovirinae*, for which numerous capsid structures have been determined (Table 1), only four crystal structures are available for *Densovirinae* (Table 5). In addition, two low resolution structures have been determined using cryo-EM (Table 5). The high-resolution structures are for *Ambidensovirus* members GmDV at 3.7 Å [7] and AdDV 3.5 Å resolution [8]; *Iteradensovirus* member BmDV1 at 3.1 Å resolution [9]; and *Penstyldensovirus* member Penaeus stylirostris densovirus (PstDV) at 2.5 Å [157]. Two of these structures, GmDV and AdDV, were determined for DNA packaged (full) infectious virions. AdDV showed three pyrimindine bases ordered within the luminal surface at the 3-fold symmetry axis [8]. As previously stated, this ordering is unexpected given the lack of icosahedral symmetry for the single copy of the packaged genome. The cryo-EM structures are for *Ambidensovirus* member *Junonia coenia* densovirus (JcDV) at 8.7 Å resolution [158] and *Brevidensovirus* member *Aedes albopictus* densovirus (AalDV2) at 15.6 Å resolution [159].

**Figure 10:**
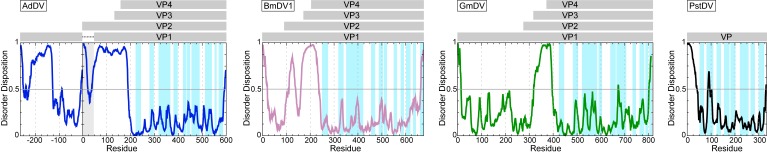
Disorder prediction for densoviruses. AdDV (blue), BmDV1 (pink), GmDV (green), and PstDV (black) VP1. The PONDR_fit application was utilized [72]. Regions above 0.5 on the Y-axis are predicted to be disordered. The approximate locations of the VPs are indicated in the grey bars above. In case of AdDV, both VP1 and VP2 have unique N-terminal regions. The regions highlighted in light blue in the disorder plot indicate the locations of the surface exposed loops, their apexes are VRs.

**Figure 11:**
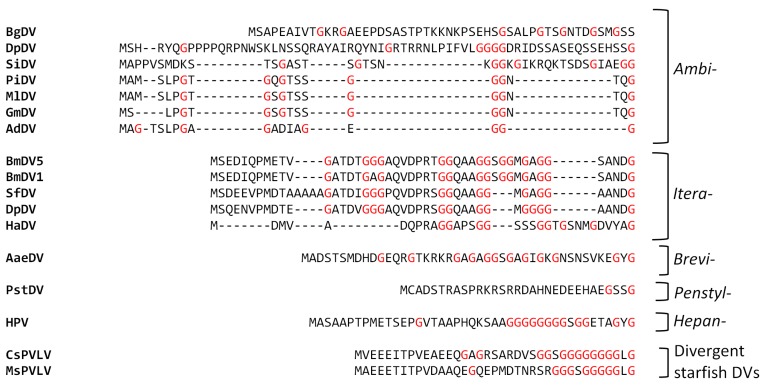
The N-termini of the major VPs of the *Densovirinae*. For each genus a selection of available VP sequences are shown for the N-terminal 20–50 amino acids. All glycine residues are in red.

Similarly to the *Parvovirinae* VP structures, a significant portion of the N-terminal region of the major capsid VP is also disordered in the densoviruses, e.g., 23 aa in GmDV and AdDV, 10 31 aa in PstDV, and 42 in BmDV1 [7,8,9,157]. Again, as for the *Parvovirinae*, disorder predictions for these viruses show disorder between the N and C-terminus at the glycine-rich region (Figure 10 and Figure 11). The glycine-rich region is significantly shorter in the densoviruses, e.g., 6 aa in GmDV and 7 aa in AdDV compared to 12 aa in CPV, but still results in a lack of structure order (Figure 10 and Figure 11). Interestingly, the BmDV1 structure currently represents the only parvoviral VP structure, where the last 40 C-terminal residues are also disordered. The C-terminal residue of densoviral VP structures, with the exception of BmDV1, are positioned near the 2-fold symmetry axis, similarly to the VP structures of the *Parvovirinae*, and exposed on the capsid surface [7,8,9,157].

**Figure 12:**
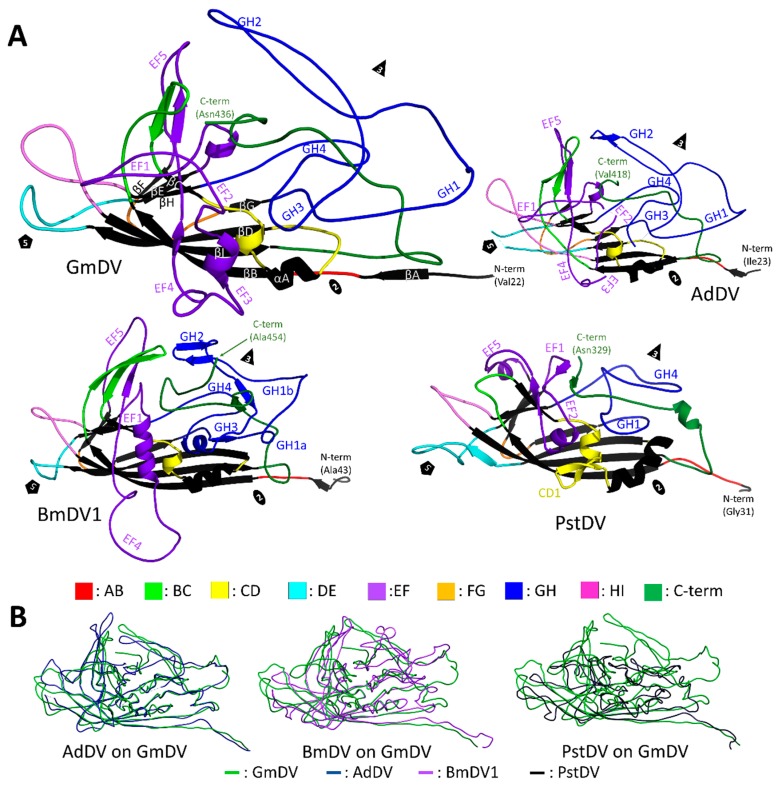
The densovirus VP structure. (**A**) Cartoon ribbon diagrams of the ordered common VP structures of GmDV and AdDV (top), BmDV1 and PstDV (bottom). The first ordered N-terminal residue and C-terminal residue are labeled. The conserved β-core and αA helix are colored in black and labeled in GmDV. Loops and subloops within the large loops are as colored in the key at the bottom and EF and GH sub-loops are labeled. The approximate 5-fold symmetry axis is marked by a pentagon, the 3-fold by a triangle, and the 2-fold by an ellipsoid. (**B**) A GmDV VP structure (*Ambidensovirus*) superimposed on the VPs of AdDV (left), BmDV1 (middle), and PstDV (right). Conformational diversity on the surface loops is evident, especially between GmDV and BmDV, and GmDV and PstDV.

The core of the densoviral VP is an eight-stranded jelly roll fold with an additional N-terminal strand, βA, and with large loops connecting the strands, as described above for the *Parvovirinae* (Figure 12). In the GmDV VP structure, considered the *Densovirinae* prototype, the EF and GH loops are further divided into five and four sub-loops, respectively (Figure 12). While the GH loop is the longest and forms most of the surface features, its length is significantly shorter compared to the corresponding loop in the *Parvovirinae* at 97 aa compared to 226 aa in CPV. Similar to the *Parvovirinae*, the GH loop is the most variable among ambidensoviruses, although VRs have not been defined, as has been done for the former viruses [7]. At the 2-fold symmetry axis, similarly to vertebrate parvovirus VPs, the densovirus structures have an alpha helix (αA). As a common feature for these viruses, a second α-helix is contained within the EF loop, with PstDV containing a third helix in the CD loop (Figure 12) [7,8,9,157].

**Figure 13:**
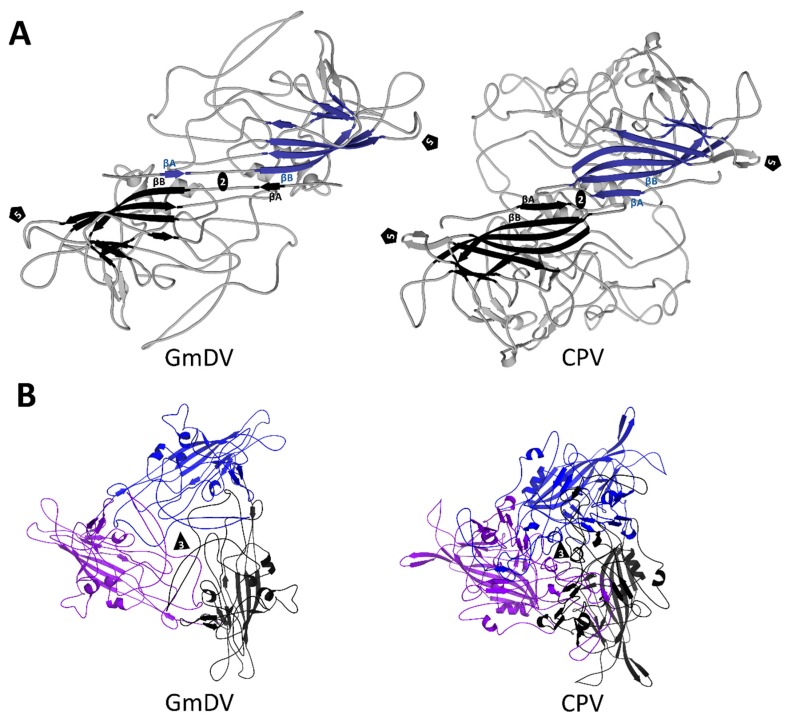
Multimeric interactions of densoviral and parvoviral VPs. (**A**) Ribbon cartoon diagrams of the interactions between βA and βB at the 2-fold symmetry axis of GmDV and CPV. The eight-stranded core, with the additional βA, which performs the domain swapping, are colored blue and black. (**B**) Interaction of three 3-fold symmetry related VPs for GmDV and CPV. The open annulus-like structure at the 3-fold axis of the densovirus trimer compared to the more closed arrangement in the vertebrate parvoviruses is evident. The triangle indicates the 3-fold axis and the pentagon the 5-fold axis.

An important and differentiating feature of the densoviral VP is the domain swapping observed at their N-terminus [7,8,9,157] (Figure 13). The βA strand of the swapped domain interacts with the 2-fold symmetry related VP’s βB strand rather than the intra VP βA and βB interaction observed in the *Parvovirinae* (Figure 13). Thus, the luminal βBIDG sheet of the jelly roll core is still extended into a βABIDG sheet, as in the *Parvovirinae*, and the first observed N-terminal residue is also positioned underneath the 5-fold axis, but in this case, under that of the neighboring VP subunit (Figure 13). In the case of the GmDV VPs, the domain swapping is proposed to create additional hydrogen bonds with the 2-fold related VP’s βB imparting increased stability [7]. A re-arrangement into an unswapped conformation is proposed to be required for VP1u externalization, although there is no experimental proof that this occurs [7]. In contrast to GmDV, in PstDV the distance between the swapped N-terminus and the βB of the neighboring VP is too large to form hydrogen bonds [157], while in AdDV, the βA contains three proline substitutions compared to GmDV, P24, P26, and P28, which makes such interactions impossible. In these two viruses, divalent cations observed at the N-terminus are hypothesized to confer stability [7,157].

The sequence identity among the VP monomers of the *Densovirinae* ranges from ~7% to 20% (Table 6). The structural similarity is higher and ranges from the value anticipated from the eight-stranded β-barrel core and αA helix at 20% to ~70% (calculated by DALI pairwise alignments [160]) between the PstDV and GmDV from different genera, and GmDV and AdDV from the same genus, respectively (Table 6). The structural diversity of densoviruses is mostly attributable to the CD, EF, and GH loops (Figure 12). In the PstDV VP structure, all loops are shorter than in the other three high-resolution structures due to the smaller size of the VP (Figure 3 and Figure 12). When the GmDV VP structure is superimposed to that of CPV, up to 148 Cα atoms (36%) are similarly positioned (not shown). This is remarkable given the lower structure similarities between members of the subfamily (Table 6). The majority of the residues are located in the core [7].

**Figure 14:**
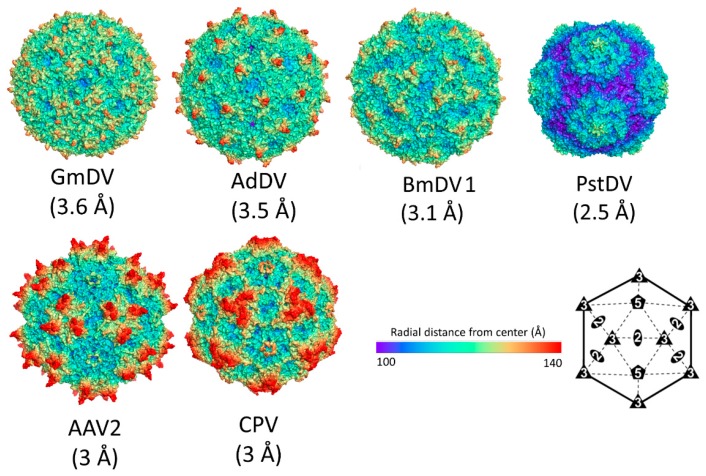
Densoviral capsid structures. The capsid surface images of GmDV, AdDV, BmDV1, and PstDV. The resolution of each structure is in parenthesis. The AAV2 and CPV capsid images are shown for comparison. The scale bar shows the radial distance (from the capsid center) used for the images. An icosahedral symmetry diagram indicating the positions of the visible symmetry axes on the capsid images are shown at the bottom right hand side.

The overall capsid morphology of densoviruses can be divided into two types: One is a large, with diameter of ~235 to ~260 Å in the depressions and protrusions, respectively, while the other one is 215 to 250 Å, being the smallest capsids so far described for the *Parvoviridae* (Figure 14). For the larger capsids, including GmDV, AdDV, and BmDV1, the capsid surface is smooth with small spike-like protrusions surrounding the 5-fold axes. In GmDV the spikes, formed by the EF4 sub-loop, appear to be smaller compared to BmDV1 and AdDV, due to the protruding GH2 sub-loop filling up the depression surrounding them. In GmDV, a smaller second protrusion is formed by the BC loop. There is a depression at the 2-fold axes (Figure 14). In the second group, containing PstDV and AalDV2 (not shown), there are prominent protrusions surrounding the 5-fold axis, forming two rim-like concentric circles. The 2- and 3-fold symmetry axes have depressions (Figure 14). Approximating with capsid size, there is a variance among luminal volume and surface area of densovirus particles consistent with the range of packaged genome (Table 7).

The 5-fold symmetry axis of the *Densovirinae*, similar to the *Parvovirinae*, contains a channel with a direct opening to the surface [7,8,9,157]. Its size is also similar, at 9 Å in diameter in GmDV, which is the same as for CPV. The inner wall of the channel is lined by large hydrophobic residues in all four structures, proposed to provide an interacting surface to the glycine-rich stretch of residues when the N-terminus is externalized. So far, the only densovirus for which PLA2 externalization has been investigated is AdDV. Meng et al. [8] found that heating of infectious AdDV particles to 70 °C resulted in increased PLA2 activity accompanied by genome ejection, while capsids remained intact. Both Simpson et al. and Meng et al. [7,8] speculated that the channel might also become occupied by stretches of VP2 and VP3 amino acids, although the role of these is currently unknown.

At the 3-fold symmetry axes, a β-annulus-like structure is present in densoviruses instead of the protrusions at or surrounding this region observed in the *Parvovirinae* (Figure 13 and Figure 14). This is similar to the 3-fold region of (+)ssRNA viruses, such as Tomato Stunt Mosaic Virus of *Tomubusviridae* [161] and Southern Bean Mosaic Virus of *Solemoviridae* [162]. The annulus is formed by charged and flexible residues, with an ~10-Å wide opening in GmDV. This opening is less pronounced in BmDV1 and shows even less annulus-like appearance in the case of PstDV, where the shorter GH loops do not interdigitate between neighboring monomers [157].

### 3.3. Functions Associated with Densoviral Capsid Proteins

Compared to members of the *Parvovirinae*, little is known about the functions of densovirus VPs and the available information is mostly based on studies of members of *Ambidensovirus*. By comparing the VP4s of two closely related lepidopteran ambidensoviruses, GmDV and JcDV, eight variable, exposed regions were identified [158]. One of these was located at the 5-fold symmetry axis, in the DE loop, five in vicinity of the protrusions surrounding it, i.e., the EF1, EF2, and the GH1 sub-loops, one at the 3-fold axis, and one in the depression at the 2-fold axis. Attempts to mutate residues in JcDV to their counterpart in GmDV showed that mutated residues in the GH loop resulted in a decrease of the ability to cross the host midgut epithelium and a reduction of JcDV virulence if introduced through the natural, gastro-intestinal pathway [163]. When *ex vivo* infecting *Spodoptera frugiperda* hosts, the mutated virus became mis-targeted and accumulated in subcellular compartments of midgut epithelial cells instead of reaching their target receptors in the basal tight junctions [164].

Recent experiments on *Blattella germanica* densovirus 1 (BgDV1) of the genus ambidensovirus have shown that an in silico predicted, bipartite nuclear localization signal (NLS) in the C-terminus of all four VPs has an effect on the import of VP monomers to the assembly site located within the nucleus. In the same viral particles a nuclear export signal (NES) was also identified that is located in the VP2 unique N-terminal region (VP2u) and was proven to function during nuclear egress of assembled BgDV1 particles. These results imply that the ambidensoviral VP2u possesses an important function for intracellular trafficking of assembled progeny virions. For this purpose the VP2u domain likely needs to be externalized, similarly to VP1u [165]. Interestingly, a functional NLS has been described at the N-terminus of hepatopancreatic parvovirus (HPV) [166]. Recently the *Helicoverpa armigera* densovirus 2 (HaDV2) VPs were shown to enhance the structural promoter activity by 35-fold compared to the activation by NS [167]. A similar role in transcriptional regulation by capsids has been proposed for the AAVs of the *Parvovirinae* [168].

## 4. Summary 

**Figure 15:**
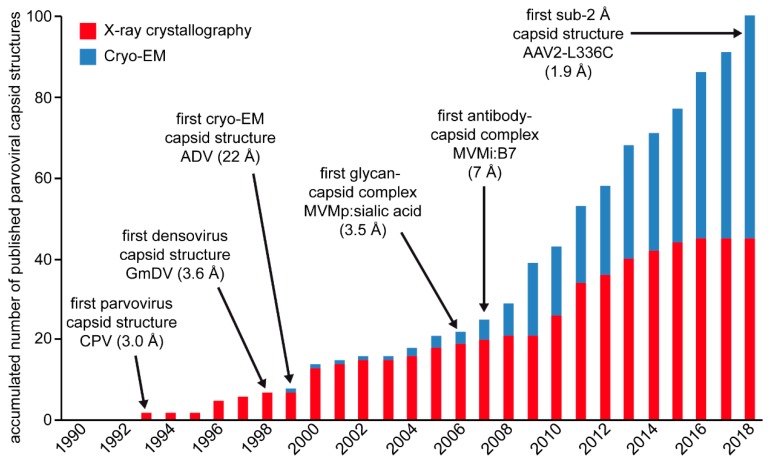
Overview of published parvoviral capsid structures since 1990. Structures determined by X-ray crystallography are shown in red and structures determined by cryo-EM in blue. Important milestones of structural parvovirology are indicated.

By the end of 2018, more than 100 capsid structures were published for the *Parvoviridae* (Figure 15). This includes lower resolution cryo-EM capsid structures alone or in complex with receptor or antibody molecules, and near-atomic and atomic resolution cryo-EM and crystal structures (Table 1, Table 3, Table 4, and Table 5). The VP coordinates for the first parvovirus capsid structure, CPV, was determined by X-ray crystallography [38]. In the following two decades, X-ray crystallography remained the method of choice to determine high-resolution capsid structures. The first parvoviral capsid structure determined by cryo-EM was for the Aleutian Mink Disease Virus (ADV) and displayed the general features of the surface of the parvoviral capsid at 22 Å resolution [169]. The first boost in the use of cryo-EM in structural parvovirology occurred after its utility for mapping the epitopes of monoclonal antibodies onto the capsid became evident. A second boost occurred with the development of direct electron detectors and their ability to record movie frames that can subsequently be aligned, which resulted in atomic resolution structures, for example, the structure of the AAV2-L336C variant at 1.86 Å resolution. This structure is currently the highest resolution parvovirus capsid structure, as well as all viruses (Figure 15). The most important aspect of the advances made in structural parvovirology is the ability to use the information obtained to functionally annotate the life cycle of these viruses. This ability provides the tools required to develop biologics in the form of vaccines or inhibitors for pathogenic members, for example, B19 and densoviruses, or gene delivery vehicles with improved efficacy for non-pathogenic members, such as the AAVs.

## Figures and Tables

**Table 1:** Summary of deposited *Parvovirinae* capsid structures.

Virus	Empty/Full	Structure Determination Method	Year	Resolution in Å	PDB-ID	Reference
***Protoparvovirus***						
BuV1	Empty	Cryo-EM	2018	2.8	6BWX	Ilyas et al. [39]
BuV2	Empty	Cryo-EM	2018	3.8	6BX0	Ilyas et al. [39]
BuV3	Empty	Cryo-EM	2018	3.3	6BX1	Ilyas et al. [39]
CPV	Empty	X-Ray Crystallography	1993	3.0	2CAS	Wu et al. [38]
CPV	Full	X-Ray Crystallography	1996	2.9	4DPV	Xie et al. [40]
CPV-N93D	Full	X-Ray Crystallography	2003	3.3	1P5Y	Govindasamy et al. [41]
CPV-N93R	Full	X-Ray Crystallography	2003	3.3	1P5W	Govindasamy et al. [41]
CPV-d-A300D	Empty	X-Ray Crystallography	2000	3.3	1C8D	Simpson et al. [42]
CPV-d-A300D	Full	X-Ray Crystallography	1996	3.3	1IJS	Llamas-Saiz et al. [43]
CPV-d pH5.5	Empty	X-Ray Crystallography	2000	3.5	1C8H	Simpson et al. [42]
CPV2a	Full	X-Ray Crystallography	2014	3.3	4QYK	Organtini et al. [44]
FPV	Empty	X-Ray Crystallography	1993	3.3	1FPV	Agbandje et al. [45]
FPV	Empty	X-Ray Crystallography	2000	3.0	1C8F	Simpson et al. [42]
FPV low pH	Empty	X-Ray Crystallography	2000	3.0	1C8G	Simpson et al. [42]
FPV no CaCl_2_	Empty	X-Ray Crystallography	2000	3.0	1C8E	Simpson et al. [42]
H-1PV	Full	X-Ray Crystallography	2013	2.7	4G0R	Halder et al. [46]
H-1PV	Empty	X-Ray Crystallography	2013	3.2	4GBT	Halder et al. [46]
LuIII	Empty	Cryo-EM	2017	3.2	6B9Q	Pittman et al. [47]
M. Spretus EVE	Empty	Cryo-EM	2018	3.9	6NF9	Callaway et al. [48]
MVMi	Full	X-Ray Crystallography	1997	3.5	1MVM	Llamas-Saiz et al. [49]
MVMi	Empty	X-Ray Crystallography	2005	3.5	1Z1C	Kontou et al. [50]
MVMi-L172W	Empty	X-Ray Crystallography	2011	4.2	2XGK	Plevka et al. [51]
MVMp	Full	X-Ray Crystallography	2005	3.3	1Z14	Kontou et al. [50]
MVMp-N170A	Empty	X-Ray Crystallography	2015	3.8	4ZPY	Guerra et al. [52]
PPV	Empty	X-Ray Crystallography	2001	3.5	1K3V	Simpson et al. [53]
***Bocaparvovirus***						
BPV	Empty	X-Ray Crystallography	2015	3.2	4QC8	Kailasan et al. [5]
HBoV1	Empty	Cryo-EM	2017	2.9	5URF	Mietzsch et al. [54]
HBoV3	Empty	Cryo-EM	2017	2.8	5US7	Mietzsch et al. [54]
HBoV4	Empty	Cryo-EM	2017	3.0	5US9	Mietzsch et al. [54]
***Dependoparvovirus***						
AAV1	Full	X-Ray Crystallography	2011	2.5	3NG9	Ng et al. [55]
AAV2	Full	X-Ray Crystallography	2002	3.0	1LP3	Xie et al. [3]
AAV2	Empty	Cryo-EM	2016	3.8	5IPI	Drouin et al. [56]
AAV2-L336C	Empty	Cryo-EM	2018	1.9	6E9D	Tan et al. [57]
AAV2-R432A	Empty	Cryo-EM	2016	3.7	5IPK	Drouin et al. [56]
AAV2.5	Full	Cryo-EM	2018	2.8	6CBE	Burg et al. [58]
AAV3	Full	X-Ray Crystallography	2010	2.6	3KIC	Lerch et al. [59]
AAV4	Full	X-Ray Crystallography	2007	3.2	2G8G	Govindasamy et al. [60]
AAV5	Empty	X-Ray Crystallography	2010	3.5	3NTT	Govindasamy et al. [61]
AAV6	Empty	X-Ray Crystallography	2010	3.0	3OAH	Ng et al. [55]
AAV6	Full	X-Ray Crystallography	2011	3.0	4V86	Xie et al. [62]
AAV8	Empty	X-Ray Crystallography	2007	2.6	2QA0	Nam et al. [63]
AAV8 pH7.5	Full	X-Ray Crystallography	2011	2.7	3RA4	Nam et al. [64]
AAV8 pH6.0	Full	X-Ray Crystallography	2011	2.7	3RA9	Nam et al. [64]
AAV8 pH5.5	Full	X-Ray Crystallography	2011	2.7	3RA8	Nam et al. [64]
AAV8 pH4.0	Full	X-Ray Crystallography	2011	2.7	3RA2	Nam et al. [64]
AAV8 pH4/7.5	Full	X-Ray Crystallography	2011	3.2	3RAA	Nam et al. [64]
AAV9	Empty	X-Ray Crystallography	2011	2.8	3UX1	Dimattia et al. [65]
AAV9-L001	Full	Cryo-EM	2019	3.2	6NXE	Guenther et al. [66]
AAV-DJ	Empty	Cryo-EM	2012	4.5	3J1Q	Lerch et al. [67]
AAVrh.8	Full	X-Ray Crystallography	2014	3.5	4RSO	Halder et al. [68]
AAVrh.32.33	Full	X-Ray Crystallography	2013	3.5	4IOV	Mikals et al. [69]
***Erythroparvovirus***						
B19	Empty	X-Ray Crystallography	2004	3.5	1S58	Kaufmann et al. [4]

**Table 2:**
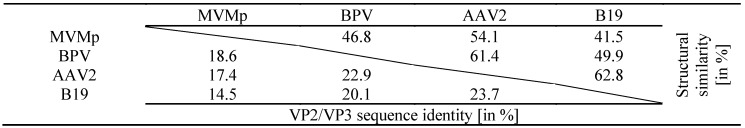
Sequence identity and structural similarity among *Parvovirinae* type members.

**Table 3:** Summary of published *Parvovirinae* capsid-receptor complex structures.

Virus	Receptor	Structure Determination Method	Year	Resolution in Å	Reference
AAV2	AAVR	Cryo-EM	2019	2.8	Zhang et al. [102]
AAV-DJ	heparinoid pentasaccharide	Cryo-EM	2017	2.8	Xie et al. [97]
AAV1	SIA	X-Ray Crystallography	2016	3.0	Huang et al. [100]
AAV5	SIA	X-Ray Crystallography	2015	3.5	Afione et al. [98]
AAV3	sucrose octasulfate	X-Ray Crystallography	2012	6.5	Lerch et al. [99]
AAV2	heparin	Cryo-EM	2009	8.3	O’Donnell et al. [95]
AAV2	heparin	Cryo-EM	2009	18.0	Levy et al. [96]
CPV	transferrin receptor	Cryo-EM	2007	25.0	Hafenstein et al. [83]
MVMp	SIA	X-Ray Crystallography	2006	3.5	López-Bueno et al. [101]

**Table 4:** Summary of published *Parvovirinae* capsid-antibody structures.

Virus	Antibody Name	Year	Binding Region	Neutralizing for Infection	Resolution in Å	Reference
***Protoparvovirus***						
CPV	Fab-E	2012	side of 3-fold protrusions across 2-fold axis	Yes	4.1	Organtini et al. [111]
CPV	Fab-14	2009	3-fold protrusions	Yes	12.4	Hafenstein et al. [84]
FPV	Fab-6	2009	3-fold protrusions	Yes	18.0	Hafenstein et al. [84]
FPV	Fab-8	2009	2/5-fold wall	Yes	8.5	Hafenstein et al. [84]
FPV	Fab-15	2009	2/5-fold wall	Yes	10.5	Hafenstein et al. [84]
FPV	Fab-16	2009	2/5-fold wall	Yes	13.0	Hafenstein et al. [84]
FPV	Fab-B	2009	3-fold protrusions	Yes	14.0	Hafenstein et al. [84]
FPV	Fab-E	2009	side of 3-fold protrusions across 2-fold axis	Yes	12.0	Hafenstein et al. [84]
FPV	Fab-F	2009	side of 3-fold protrusions across 2-fold axis	Yes	14.0	Hafenstein et al. [84]
MVMi	B7	2007	center of 3-fold symmetry axis	Yes	7.0	Kaufmann et al. [112]
***Bocaparvovirus***						
HBoV1	4C2	2016	3-fold protrusions	unknown	16.0	Kailasan et al. [113]
HBoV1	9G12	2016	3-fold protrusions	unknown	8.5	Kailasan et al. [113]
HBoV1	12C1	2016	3-fold protrusions	unknown	11.9	Kailasan et al. [113]
HBoV1	15C6	2016	around 5-fold symmetry axis	unknown	18.6	Kailasan et al. [113]
HBoV2	15C6	2016	around 5-fold symmetry axis	unknown	17.8	Kailasan et al. [113]
HBoV4	15C6	2016	around 5-fold symmetry axis	unknown	9.5	Kailasan et al. [113]
***Dependoparvovirus***						
AAV1	ADK1a	2015	3-fold protrusions	Yes	11.0	Tseng et al. [114]
AAV1	ADK1b	2015	2/5-fold wall	Yes	11.0	Tseng et al. [114]
AAV1	4E4	2013	side of 3-fold protrusions across 2-fold axis	Yes	12.0	Gurda et al. [115]
AAV1	5H7	2013	center of 3-fold symmetry axis	Yes	23.0	Gurda et al. [115]
AAV2	C37-B	2013	3-fold protrusions	Yes	11.0	Gurda et al. [115]
AAV2	A20	2012	2/5-fold wall	Yes	8.5	McCraw et al. [116]
AAV5	ADK5a	2015	2/5-fold wall	Yes	11.0	Tseng et al. [114]
AAV5	ADK5b	2015	2/5-fold wall to 5-fold symmetry axis	Yes	12.0	Tseng et al. [114]
AAV5	HL2476	2018	3-fold protrusions	Yes	3.1	Jose et al. [108]
AAV5	3C5	2013	2/5-fold wall in a tangential orientation	No	16.0	Gurda et al. [115]
AAV6	5H7	2013	center of 3-fold symmetry axis	unknown	15.0	Gurda et al. [115]
AAV6	ADK6	2018	3-fold protrusions & 2/5-fold wall	Yes	13.0	Bennett et al. [117]
AAV8	ADK8	2011	3-fold protrusions	Yes	18.7	Gurda et al. [118]
AAV9	PAV9.1	2018	center of 3-fold symmetry axis	Yes	4.2	Giles et al. [119]
***Erythroparvovirus***						
B19	human Fab	2018	around 5-fold symmetry axis	Yes	3.2	Sun et al. [120]

**Table 5:** Capsid structures of *Densovirinae* determined to date.

Virus	Empty / Full	Structure Determination Method	Year	Resolution in Å	PDB-ID	Reference
***Ambidensovirus***						
AdDV	Full	X-Ray Crystallography	2013	3.5	4MGU	Meng et al. [8]
GmDV	Full	X-Ray Crystallography	1998	3.6	1DNV	Simpson et al. [7]
JcDV	Empty	Cryo-EM	2005	8.7	N/A	Bruemmer et al. [158]
***Brevidensovirus***						
AalDV2	Full	Cryo-EM	2004	15.6	N/A	Chen et al. [159]
***Iteradensovirus***						
BmDV1	Empty	X-Ray Crystallography	2011	3.1	3P0S	Kaufmann et al. [9]
***Penstyldensovirus***						
PstDV	Empty	X-Ray Crystallography	2010	2.5	3N7X	Kaufmann et al. [157]

**Table 6:**
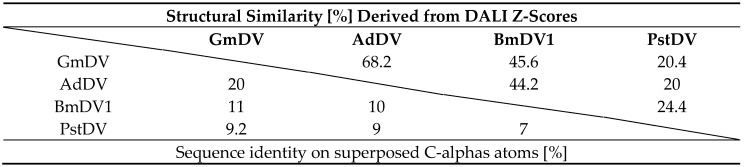
Sequence identity and structural similarity for densoviruses.

**Table 7:** Dimensions, DNA content, and taxonomy of densovirus capsid structures.

	Inner Radius (Å)	Inner Surface Area (nm^2^)	Inner Volume (nm^3^)	Genome Size (nt)	Genus
GmDV	98.7	1223.2	4022.6	6039	*Ambidensovirus*
AdDV	91.7	1056.2	3227.8	5425	*Ambidensovirus*
BmDV1	98.7	1224	4027.5	5076	*Iteradensovirus*
PstDV	87.6	963.3	2811.5	3914	*Penstyldensovirus*
CPV	92.9	1084.8	3359.5	5323	*Protoparvovirus*
AAV2	89.9	1014.9	3040.4	4679	*Dependoparvovirus*
